# Glucose 6-Phosphate Dehydrogenase from Trypanosomes: Selectivity for Steroids and Chemical Validation in Bloodstream *Trypanosoma brucei*

**DOI:** 10.3390/molecules26020358

**Published:** 2021-01-12

**Authors:** Cecilia Ortíz, Francesca Moraca, Marc Laverriere, Allan Jordan, Niall Hamilton, Marcelo A. Comini

**Affiliations:** 1 Redox Biology of Trypanosomes, Institut Pasteur de Montevideo, Mataojo 2020, Montevideo 11400, Uruguay; cortiz@pasteur.edu.uy; 2 Dipartimento di Biotecnologie, Chimica e Farmacia, Università degli Studi di Siena, Via Aldo Moro 2, 53100 Siena, Italy; francesca.moraca@gmail.com; 3 Instituto de Investigaciones Biotecnológicas, Instituto Tecnológico de Chascomus (IIB-INTECH, UNSAM-CONICET), Av. General Paz 5445, INTI, San Martín 1650, Pcia de Buenos Aires, Argentina; marc.laverriere@pasteur.fr; 4 Drug Discovery Unit, Cancer Research UK Manchester Institute, University of Manchester, Alderley Park, Macclesfield SK10 4TG, UK; a.jordan@sygnaturediscovery.com (A.J.); niall.hamilton@tiscali.co.uk (N.H.)

**Keywords:** androstane, redox, *Trypanosoma brucei*, *Trypanosoma cruzi*, pentose phosphate pathway

## Abstract

Glucose 6-phosphate dehydrogenase (G6PDH) fulfills an essential role in cell physiology by catalyzing the production of NADPH^+^ and of a precursor for the de novo synthesis of ribose 5-phosphate. In trypanosomatids, G6PDH is essential for in vitro proliferation, antioxidant defense and, thereby, drug resistance mechanisms. So far, 16α-brominated epiandrosterone represents the most potent hit targeting trypanosomal G6PDH. Here, we extended the investigations on this important drug target and its inhibition by using a small subset of androstane derivatives. In *Trypanosoma cruzi*, immunofluorescence revealed a cytoplasmic distribution of G6PDH and the absence of signal in major organelles. Cytochemical assays confirmed parasitic G6PDH as the molecular target of epiandrosterone. Structure-activity analysis for a set of new (dehydro)epiandrosterone derivatives revealed that bromination at position 16α of the cyclopentane moiety yielded more potent *T. cruzi* G6PDH inhibitors than the corresponding β-substituted analogues. For the 16α brominated compounds, the inclusion of an acetoxy group at position 3 either proved detrimental or enhanced the activity of the epiandrosterone or the dehydroepiandrosterone derivatives, respectively. Most derivatives presented single digit μM EC_50_ against infective *T. brucei* and the killing mechanism involved an early thiol-redox unbalance. This data suggests that infective African trypanosomes lack efficient NADPH^+^-synthesizing pathways, beyond the Pentose Phosphate, to maintain thiol-redox homeostasis.

## 1. Introduction

Chagas disease, African trypanosomiasis and leishmaniasis are “neglected tropical diseases” (NTD) that affect the most vulnerable populations around tropical and subtropical regions of the world. More than 100 million persons suffer from trypanosomiasis and leishmaniasis, and several thousand die annually [1] due to difficulties in access to treatments that, in terms of efficacy and safety, are far from ideal [2,3]. These are zoonotic diseases caused by digenetic protozoan (Familiy Trypanosomatidae) that evolved several unusual biochemical and structural characteristics to overcome the challenging environmental conditions imposed by their complex life cycles [4]. In this context, components of key metabolic pathways eral enzymes producing or depending on NADPH have raised interest as promising drug targets against trypanosomatids [5,6,7,8,9]. NADPH is an essential molecule that provides reducing power for the biosynthesis and repair of macromolecules, as weare very attractive for the development of potent and selective drugs. Among them, sevll as for the detoxification of xenobiotics and different reactive species derived from partial reduction of oxygen or from nitric oxide.

Several enzymes may contribute to the production of NADPH in different subcellular compartments of the infective stages of trypanosomatids [10,11]. They can be grouped in dehydrogenases participating in amino acid (isocitrate dehydrogenase: IDH, malate dehydrogenase: MDH, glutamate dehydrogenase: GDH) or carbohydrate metabolism (glucose 6-phosphate dehydrogenase: G6PDH and 6-phosphogluconate dehydrogenase: 6PGDH). Such redundancy enables the parasite to maintain the NADPH pool under different growth conditions. For instance, in glucose-rich media (e.g., mammalian bloodstream, lymph and cerebrospinal fluid), African trypanosomes rely on glucose 6-phosphate oxidation by enzymes (i.e., G6PDH and 6PGDH) that belong to the oxidative branch of the pentose phosphate pathway (PPP) to synthesize NADPH [11]. Under glucose starvation, the flux through the oxidative branch of the PPP is reduced and NADPH homeostasis is mainly sustained by a transhydrogenase-like shunt involving cytosolic and mitochondrial MDH [11]. Nonetheless, a significant amount of cytosolic NADPH has been shown to be produced by the PPP (ca. 25% in bloodstream *T. brucei*) due to gluconeogenesis fueled by glycerol (in the mammalian stage) [12] or proline (in the insect stage) [13]. G6PDH [14] and 6PGDH [15] proved indispensable for the bloodstream form of *T. brucei*, though it remains unclear whether this is a consequence of a shortage of NAPDH and/or ribose 5-phosphate. For *T. cruzi*, several NADP-dependent dehydrogenases have been characterized [16,17,18,19,20,21,22] but a quantitative assessment of their contribution to NADPH homeostasis in the infective stages has not yet been performed. In the mammalian host and at variance with *T. brucei*, *T. cruzi* can colonize different host cells and obtain reducing equivalents from amino acid metabolism via the Krebs’ cycle [23]. Nonetheless and independently of the cell cycle stage, the oxidative branch of the PPP appears to efficiently back up the NADPH demand of *T. cruzi* upon oxidative challenge. In fact, treatment of parasites with H_2_O_2_ [19] or the redox cycling drug methylene blue upregulated the expression of G6PDH and the rate of glucose consumption through the PPP [24,25], respectively.

Due to its house-keeping role in metabolism, G6PDH has raised special interest as a drug target candidate to treat trypanosomal [11,26] and non-communicable human diseases, such as cancer [27,28]. Among the molecules with potential to inhibit G6PDH, the steroid derivatives represent the first and best characterized inhibitors of the human [27,29,30,31] and pathogen enzyme [14,32,33,34]. (Dehydro)epiandrosterone derivatives were described as anti-competitive inhibitors of G6PDH [14] whose inhibitory potential and selectivity can be tuned by including specific substituents at positions 3 or 16 [27,33]. Taking advantage of the availability of crystal structures for the human [35,36,37] and *T. cruzi* G6PDH [22,38], the potential binding site of steroids to these enzymes have been investigated. In silico studies performed on the structure of the binary G6PDH-G6P complex suggested that steroids partially occupy the NADP+ binding site of the human enzyme [39], which in part conflicts with the proposed uncompetitive inhibition kinetics described for these compounds. In contrast, and according to data arising from complementary computational and biochemical analysis, the steroids were proposed to bind to *T. cruzi* G6PDH in a region next to the G6P-binding pocket and close to the catalytic site [33].

The metabolic relevance of G6PDH and its selective drugability by steroids encouraged us to characterize the subcellular localization of the protein in a pathogen parasite, to demonstrate the on-target/mode of killing of **EA** and **DHEA** derivatives in infective trypanosomes and to provide some clues on the molecular determinants for the differential binding mode of these compounds to the host and parasite enzyme. In the present study, all of these questions are subjects of investigation.

## 2. Results and Discussion

### 2.1. G6PDH Is a Low Abundant and, Predominantly, Cytosolic Protein in Non-Infective T. cruzi

**Figure 1:**
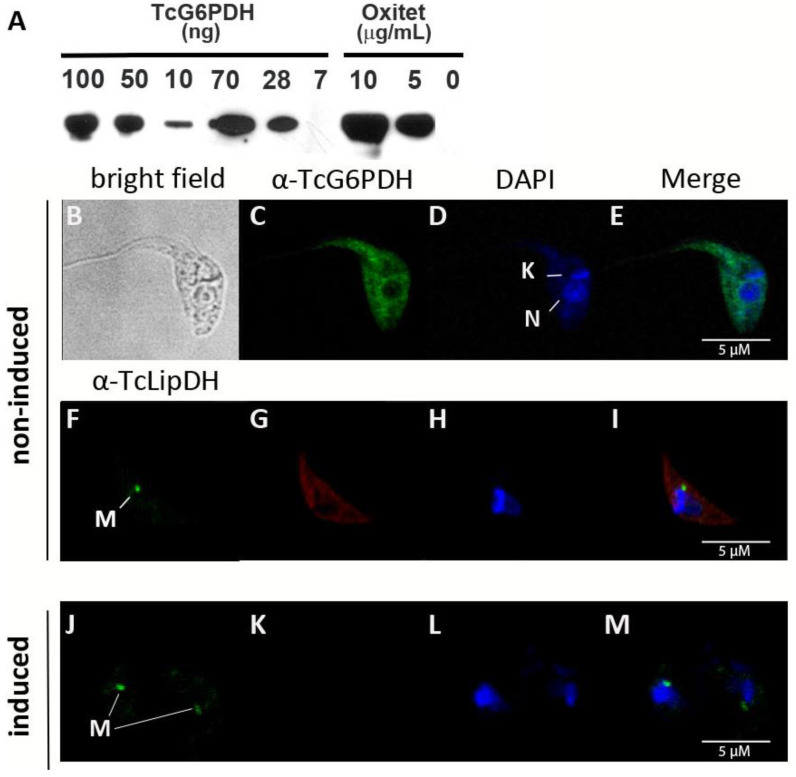
Subcellular distribution of G6PDH in *T. cruzi* epimastigotes (strain Adriana). (**A**) Western blot analysis of different amounts of recombinant *Tc*G6PDH (7, 10, 28, 50, 70 and 100 ng) and cell extracts of oxytetracycline induced (5 or 10 μg/mL: 1 × 10^7^ cells/lane) and non-induced (0 μg/mL: 1 × 10^6^ cells/lane) trypanosomes. Confocal microscopy of samples from induced (**B**–**I**) and non-induced (**J**–**M**) parasites. (**C**,**G**,**K**) Indirect immunofluorescence using anti-*Tc*G6PDHL and secondary anti-mouse serum conjugated to Alexa 488 (green signal) or Alexa 594 (red signal), respectively. (**D**,**H**,**L**) Mitochondrial (**K**) and nuclear (**N**) DNA was stained with DAPI (blue signal). (**F**,**J**) signal corresponding to the mitochondrial lipoamide dehydrogenase (**M**) detected with specific anti-serum against the *T. cruzi* protein. (**E**,**I**,**M**) Merge images of the fluorescence staining.

Specific polyclonal antibodies against TcG6PDHL were raised in mice [22] and used for Western blot and immunofluorescence analysis in the insect stage of *T. cruzi*. In line with previous observations [18], immune-detection (blotting or microscopy) of endogenous G6PDH proved unsuccessful in epimastigote parasites (Figure 1A), which confirms that G6PDH is a low abundant protein in the non-infective stage of the parasite.

In order to overcome this limitation and estimate the intracellular concentration and subcellular localization of the protein, a tetracycline-inducible ectopic copy of TcG6PDHL was expressed in *T. cruzi*. As shown in Figure 1A, G6PDH became detectable in total lysates from transgenic parasites cultured in the presence of oxytetracycline but not in its absence. Based on the detection limit of the antibodies (up to 10 ng TcG6PDHL), the total cell number in the analyzed lysate and the cell volume reported for *T. cruzi* epimastigotes (30 fL) [40], we estimated that the intracellular concentration of G6PDH in this parasite species and stage is lower than 0.7 μM.

Immunofluorescence using the anti-G6PDH serum and secondary antibodies labeled with different fluorophores revealed a predominantly cytosolic distribution of the protein in *T. cruzi* from the Adriana (Figure 1B,D) and CL-Brener strain (Figure S1). The G6PDH signal did not overlap with that obtained for the nucleus or the mitochondrial protein lipoamide dehydrogenase (Figure 1C,E) [41,42]. Unfortunately, we were unable to detect G6PDH in total cell lysates of *T. brucei*, even when using a higher concentration of TcG6PDH anti-serum (not shown), which can be ascribed to the lack of strong cross-reactivity of the antibodies or to a lower intracellular content of this protein in African trypanosomes.

**Figure 2:**
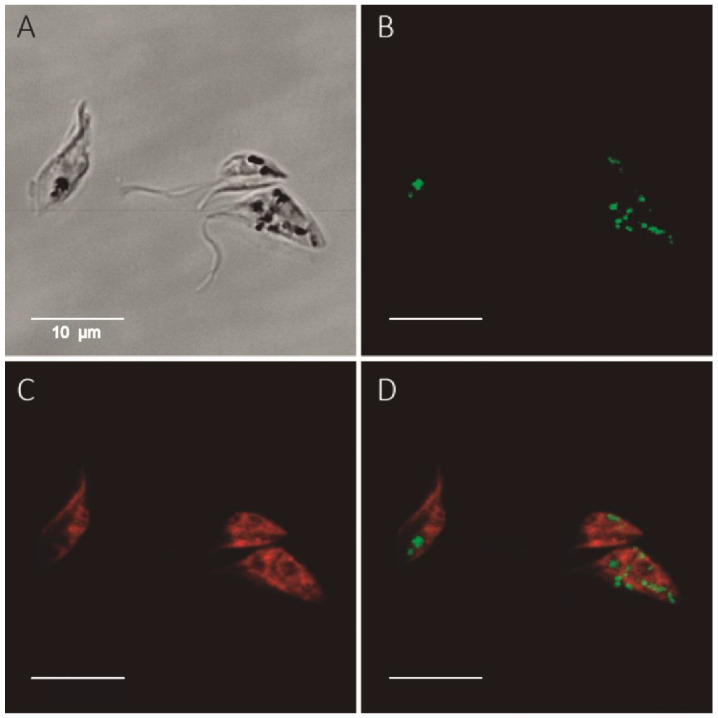
In situ detection of G6PDH activity in *T. cruzi* overexpressing G6PDH. Epimastigotes (*T. cruzi* strain Adriana) were grown for 24 h in the presence of 10 μg/mL oxytetracycline to induce overexpression of *Tc*G6PDHL (Western blot shown in Figure 1A). The formazan fluorescent signal (green color) generated by the NADPH-dependent reduction of 5-cyano-2,3-ditolyl-tetrazolium chloride corresponds to G6PDH activity. (**A**) Bright field image with light dense intracellular particles corresponding to precipitated formazan salt. (**B**) Fluorescent formazan signal. (**C**) Cell membranes stained with CellMask. (**D**) Merged image of the fluorescence staining.

To further confirm these results, we employed a cytochemical assay where the in situ production of NADPH by G6PDH is coupled to the formation of fluorescent formazan salts via the electron carrier methoxyphenazine methosulphate [43]. In order to ensure the specificity of the NADPH source, mitochondrial energetic metabolism is shut-off through the addition of sodium azide and G6P/NADP^+^. As shown in Figure 2, a formazan signal was confined to parasite cytosol of G6PDH overexpressing parasites and was fully absent in samples where G6P was omitted as a substrate (Figure S1A–D). This assay also allowed the detection of the cytosolic signal from the endogenous G6PDH activity in non-induced parasites (Figure S1E).

### 2.2. Epiandrosterone (EA) Targets G6PDH in T. cruzi

The only and indirect evidence showing that steroids target G6PDH at the cellular lever stems from assays performed on a *T. brucei* cell line that was genetically engineered to express the *Leishmania mexicana* G6PDH [32]. Unlike the trypanosomal G6PDH, *L. mexicana*, G6PDH is refractory to **EA** and **DHEA** inhibition [14], hence *T. brucei* expressing the leishmanial enzyme was resistant to these steroids.

**Figure 3:**
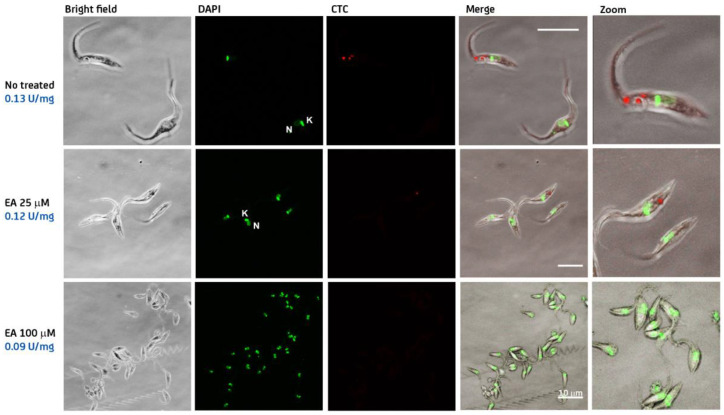
In situ detection of G6PDH activity in *T. cruzi* strain CL Brenner. Epimastigotes were incubated for 24 h in the absence (no treated) or presence of 25 and 100 μM epiandrosterone (**EA**). G6PDH activity was measured in cell extracts using the standard enzyme assay (specific activity expressed as U/mg) and detected at intracellular level using a couple cytochemical assay based on the reduction of 5-cyano-2,3-ditolyl-tetrazolium chloride (CTC; red color). Hoechst 3342 (green color) was used to stain nuclear and mitochondrial DNA. Bright field and merged images are also shown with the white bar indicating a length of 10 μm.

In order to provide direct evidence for the on-target effect of steroids in *T. cruzi*, epimastigotes from different strains (CL-Brener and Dm28c) were treated for 24 h with different concentrations of **EA** (0–100 µM) and G6PDH activity was measured in the supernatant of cell lysates and in situ using the cytochemical assay described above. For both *T. cruzi* strains, the specific G6PDH activity measured in cell extracts decreased in a concentration-dependent manner with respect to **EA** (Figure 3 for strain CL-Brener and Figure S2 for Dm28C). Similarly, the cytochemical assay revealed a discrete fluorescence staining of the cytosol for untreated parasites that decreased in intensity or was absent in samples treated with increasing concentrations of **EA** (Figure 3 and Figure S2). The percentage of cells displaying positive signal for G6PDH activity at the different **EA** concentrations is shown in Figure S2.

These results strongly suggest that the major molecular target of **EA** in *T. cruzi* is G6PDH.

### 2.3. Inhibition of G6PDH by New (Dehydro)Epiandrosterone Derivatives

**Figure 4:**
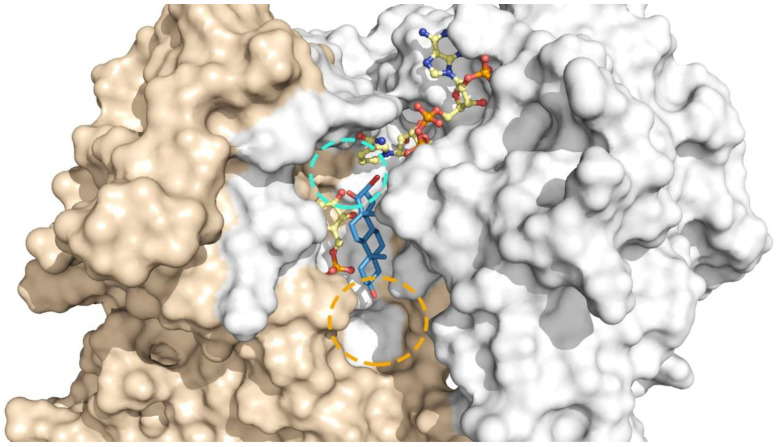
Docking of 16α-bromo-epiandrosterone to *T. cruzi* G6PDH. Catalytic model of TcG6PDH (NADP^+^ and G6P are shown in yellow sticks) with bound bromo-epiandrosterone (blue sticks) [33]. The N-terminal Rossmann-fold and the C-terminal dimerization domain of TcG6PDH are shown as surface colored in light-gray and wheat color, respectively. The protein pockets and positions of the steroids that are suited for substitutions are circled in cyan and orange.

According to previous molecular and biochemical studies [33], substitutions at the cyclopentane ring and position 3 of steroids can fine-tune the inhibitory potential of these molecules against TcG6PDH (Figure 4). For instance, bromination of **EA** and **DHEA** at position 16 yielded very potent and selective TcG6PDH inhibitors [14]. However, the relevance of the halogen orientation (α or β) for enzyme inhibition has not yet been addressed. On the other hand, polar substituents were well tolerated at position 3, yielding slightly better inhibitors of TcG6PDH than unsubstituted **EA** [33].

**Figure 5:**
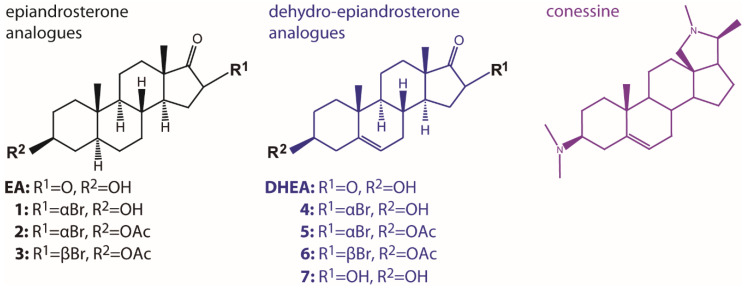
Steroid analogues tested in this study.

Nonetheless, the role of apolar substituents at position 3 was not explored. Thus, here we addressed these questions by investigating the performance of a small subset of 3-acetoxy, 16α and 16β brominated **EA** and **DHEA** derivatives towards the recombinant form of the pathogen and human G6PDH (Figure 5). Conesine, a steroid alkaloid containing a pyrrole ring at the pentane moiety and with very potent anti-*T. brucei* activity [44], was also analyzed.

In agreement with a previous report [14], **DHEA** and **EA** displayed a similar inhibitory activity towards human G6PDH (IC_50_ ~10 µM), whereas the latter was 7-fold more active against TcG6PDH (IC_50_ = 3 µM; Table 1). Interestingly, the inclusion of an H-bond donor group (OH) at position 16 of **DHEA** did not significantly modify the potency towards the human enzyme (**7**, IC_50_ = 15.8 µM) but instead proved to be detrimental for inhibition of TcG6PDH (**7**, IC_50_ > 50 µM). Interestingly, 16α-bromination of the cyclopentane moiety had profound effects on the potency and selectivity of the steroids towards the molecular targets. Compared to the parent molecules, 16α-BrEA (**1**, IC_50_ = 0.053 µM) and 16α-BrDHEA (**4**, IC_50_ = 3.3 µM) showed a 56- and 7-fold higher levels of inhibitory activity against TcG6PDH, respectively. Conversely, against the human enzyme both brominated derivatives were 2- to 4-fold (IC_50_ = 20.8 and 48.0 µM for 16α-BrEA and 16α-BrDHEA, respectively) less active than the unmodified steroids. A similar selectivity towards TcG6PDH was reported for 16BrEA [26]. Thus, halogenation of **EA** and **DHEA** increased the selectivity of the steroids for the pathogen vs. the host enzyme by 130- and 28-fold, respectively.

The inclusion of an apolar acetoxy group at position 3 of 16α-brominated **EA** (2) and **DHEA** (5) almost abrogated the inhibition of human G6PDH (0% inhibition at 100 µM compounds). With respect to TcG6PDH and when compared to the 3-unmodified molecules, the acetoxy group proved to be slightly detrimental for the activity of 16α-BrEA (**2**, IC_50_ = 0.11 µM) but significantly enhanced activity for 16α-BrDHEA (**5**, IC_50_ = 0.079 µM). Indeed, the loss of human G6PDH inhibition conferred by the incorporation of the acetoxy group along with the submicromolar activity against the pathogen enzyme resulted in both compounds showing the highest selectivity towards TcG6PDH (>900) ever reported.

Notably, for the acetoxy derivatives the orientation of the halogen atom appears to be a key determinant for the anti-TcG6PDH activity, since the **EA** and **DHEA** analogues harboring a β-Br were 13- (**3**, IC_50_ = 1.5 µM) and >600-fold (**6**, IC_50_ > 50 µM) less active than the α-Br analogues (**2** and **4**, respectively). Against the human enzyme and similar to the α-Br analogues, both β-brominated 3-acetoxy steroids proved to be inactive at 100 µM, indicating that the acetoxy group to a great extent affects the binding of the steroids to the host protein.

At the highest concentration tested (100 µM), Conesine did not inhibit G6PDH from both species, which demonstrated that this is not its molecular target.

### 2.4. Differential Binding Mode of Steroids to Human and T. cruzi G6PDH

**Figure 6:**
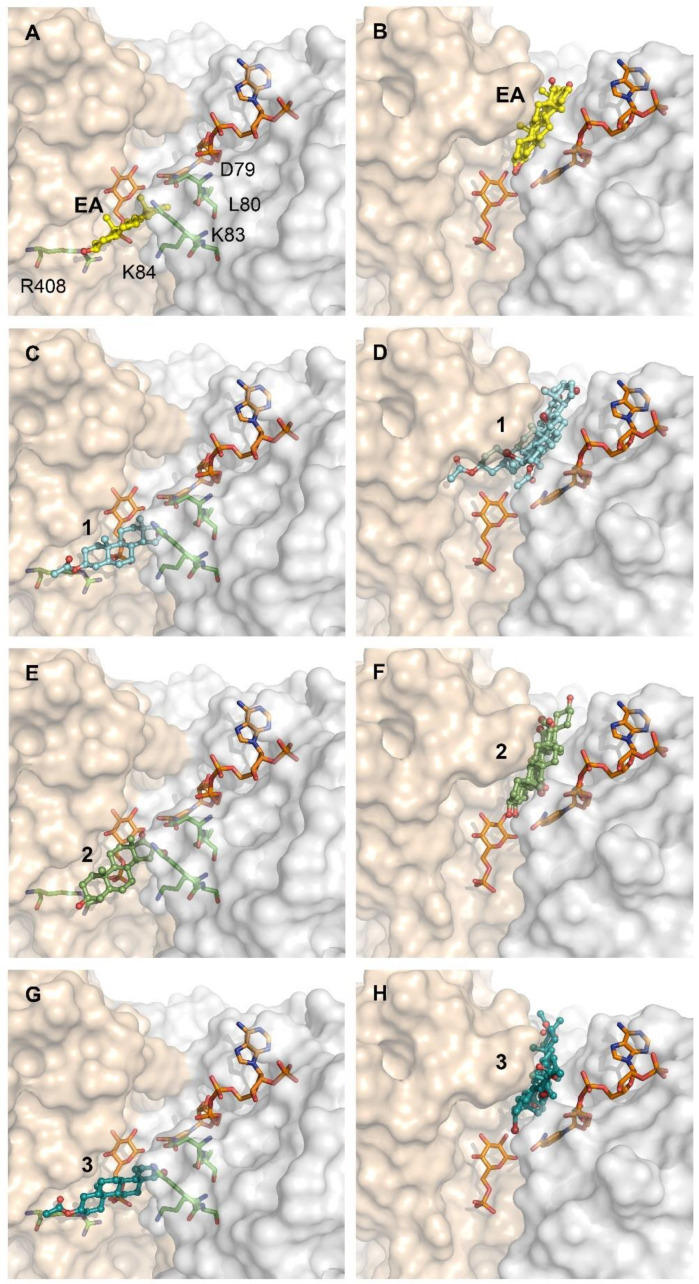
Docking of steroids derivatives to *T. cruzi* and human G6PDH. Catalytic model of TcG6PDH (left side) and HsG6PDH (right side), NADP^+^ and G6P are shown in orange sticks. (**A**,**B**) with bound epiandrosterone (yellow balls and sticks), (**C**,**D**) compound **1** (cyan balls and sticks), (**E**,**F**) compound **2** (green smudge balls and sticks), (**G**,**H**) compound **3** (deep teal balls and sticks). The N-terminal Rossmann-fold and the C-terminal dimerization domain of TcG6PDH is shown as surfaces colored in light-gray and wheat color, respectively. Residues involved in steroid binding to TcG6PDH: D79, L80, K83, K84 and R408 are depicted with green sticks in (**A**,**C**,**E**,**G**).

The remarkable selectivity of the new acetoxy derivatives of 16α-BrEA or **DHEA** is suggestive of major differences in the binding mode/affinity of these compounds for the human and parasite enzyme. In order to provide some preliminary clues towards this question, the potential binding mode of the **EA** analogues to *T. cruzi* and human G6PDH was modeled using computational approaches (Figure 6). Based on the fact that steroids inhibit G6PDH from both species by an anticompetitive mechanism [14,31], the analysis was performed for each enzyme-substrates complex. In this study, we saved three docking poses per ligand and we chose the best one based on the docking score combined with how many times a pose would be duplicated among the three saved as an indication of a higher consistency (and perhaps stability) of the pose itself. Indeed, two out of three poses were found to be overlaid in the docking performed on *T. cruzi* G6PDH for all compounds. For the human G6PDH, two out of three poses only overlay for EA, 16α-BrEA and 16β-BrEA. Compounds **2** and **3** show a very different binding mode in all the three poses saved, indicating that none of the contacts is favorable enough to stabilize a pose.

In agreement with our previous studies (Figure 4) [33], the binding site of the new steroids to TcG6PDH lies in a pocket beneath that of G6P (Figure 6A,C,E,G). The cyclopentanone moiety of **EA** shows hydrophobic contacts with Y295, the cyclohexane in position 13-14 with the nicotinamide ring of NAP^+^ and the methyl group in position 10 with I287. For 16α-BrEA (1), its cyclohexane in position 5 makes additional contacts with K83 and the Br loses a contact with the nicotinamide ring, but maintains the ones with the NAP^+^ phosphate group and Y295. This slight reorganization in the protein-inhibitor contact and the steric effect of Br in the catalytic site may explain the higher potency of the brominated derivative. In line with their similar inhibitory activity towards TcG6PDH, the acetoxy derivative **2** shows a binding mode consistent with that of 16α-BrEA. For compound **2**, the methyl group in position 13 is oriented parallel to the G6P binding site and the Br oriented towards the NAP^+^, making hydrophobic contacts with the nicotinamide group, the phosphate group and also Y295. The cyclopentane makes hydrophobic interactions with Y295 as well, and the cyclohexane in position 13–15 interacts with the nicotinamide moiety of NAP^+^. The methyl group in position 10 makes hydrophobic contacts with I287. Overall, the orientation of the steroidal rings for compound **3** is similar to that of compound **2**; however, the β-Br atom is now oriented towards the G6P binding site and the number of hydrophobic contacts is reduced. Specifically, the halogen atom shows contacts with only Y295; the cyclopentane does not show any contact with the enzyme nor the cofactor; the cyclohexane only shows contacts with the nicotinamide moiety of NAP^+^ and the methyl group in position 10 maintains contacts with I287. This may explain the comparatively lower activity of compound **3** with respect to **1** and **2** (µM vs. nM; Table 1).

Interestingly, the binding site of steroids to human G6PDH suggested by our analysis is very similar to that reported previously [39], except for the orientation adopted by the ligands that is shifted almost 90° in the plane of the steroid rings. In fact, whereas in the model generated on the G6P-HsG6PDH complex the representative steroids partially occupied the substrate binding sites [39], our model shows that **EA** and its 16α-Br derivative do not interfere with substrate binding and are tightly embedded in a hydrophobic pocket formed by the apolar side chains of Pro143, Pro144, Pro172 and Tyr249, and are located near the active site (Figure 6B,D). In the case of **EA**, our model shows that its cyclopentanone moiety makes hydrophobic contacts with P144 and P143; the cyclohexane in position 13-14, the methyl group in position 10 and the cyclohexane in position 10-5 are making hydrophobic contacts with F253. The cyclohexane in position 10-5 is in contact with the nicotinamide moiety of NADP^+^ as well. The methyl group in position 13 makes hydrophobic contacts with Y249. For 16α-BrEA, the halogen atom makes hydrophobic contacts with E252 and F253; similar to **EA**, the cyclopentanone interacts with P144 and P143; the cyclohexane in position 13-14 makes hydrophobic contacts with F253 and Y249; both methyl groups are oriented towards the NADP^+^ and the one in position 10 makes hydrophobic contacts with the nicotinamide moiety of NADP^+^ together with the cyclohexane in position 10-5.

As commented on above, docking of **2** and **3** into HsG6PDH yielded poses that differed markedly between each other (rotations of 90° and 180°) in the orientation of the steroid rings (and their methyl groups) and, consequently, affected the 3′-acetoxy substitution. This unstable binding is consistent with the experimental data (Table 1), namely that these compounds do not inhibit the human G6PDH, and can be attributed to the acetoxy group.

**Figure 7:**
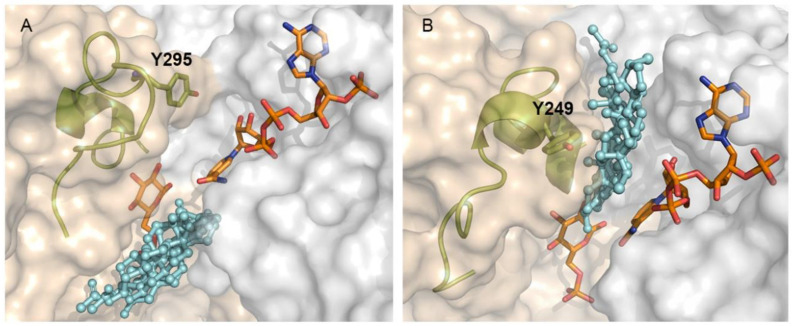
Differential binding site of steroids derivatives to *T. cruzi* and human G6PDH. (**A**) Catalytic model of TcG6PDH (left side) and (**B**) HsG6PDH (right side), NADP^+^ and G6P are shown as orange sticks. Steroid derivatives (cyan balls and sticks). The element containing the tyrosine residue (Y295 and Y249 in Tc- and Hs-G6PDH) is shown as cartoons and olive colored sticks. The N-terminal Rossmann-fold and the C-terminal dimerization domain of TcG6PDH are shown as having their surfaces colored in light-gray and wheat color, respectively.

The different binding sites of steroids to human and *T. cruzi* G6PDH raises a question about the structural determinant of the protein behind this selectivity. As reported previously, the active and substrate binding site of both proteins is to a large extent very similar [22,38]. However, a closer inspection reveals that the region corresponding to the binding pocket for steroids in the human enzyme is partially occluded by Y295 in the parasite enzyme (Figure 7A). Y295 belongs to the C-terminal dimerization domain of the protein and is located in a largely disordered region that allows the lateral chain of this residue to face and make contact with residues from the Rossmann-fold domain. Furthermore, the looser conformation of this region, which can be seen as a lid over the G6P binding site, allows G6P to locate deeper into the active site and consequently to free space in a pocket accessible to the solvent, where the steroids bind. In contrast, the equivalent residue of human G6PDH, namely Y249, is located in a well packed α-turn and facing the solvent. In this conformation, a narrow cleft is formed between both protein domains, which allows for sandwiching the steroids in the human enzyme. The narrowness of the steroid binding site in the human enzyme, which imposes important steric restrictions to the ligands, may also explain why inhibition of the human enzyme by certain steroids proves to be more difficult than TcG6PDH.

### 2.5. Biological Activity of Steroid Derivatives

The biological activity of the compounds was tested against epimastigotes of *T. cruzi*, bloodstream *T. brucei brucei* and murine macrophages (cell line J774).

None of the compounds caused a significant cytotoxic effect on *T. cruzi* when tested at 1 and 10 µM for 72 h (not shown). At 50 µM (Table 2), the brominated and acetoxy-substituted analogues (**2**, **3**, **5** and **6**) proved to be significantly more active (cytotoxicity 30–76%) than the related derivatives lacking one (**1**, **4**) or both substitutions (**EA**, **7**; cytotoxicity 4–15%). The acetoxy analogues of **EA** and **DHEA** with the Br atom in β orientation displayed the highest cytotoxic effect (**3**: 74% and **6**: 76%). Strikingly, the most potent inhibitor of TcG6PDH, compound **1** (IC_50_ 53 nM), affected cell viability to a minor extent (15%) when compared to compound **6** (cytotoxicity of 76%), which has a 1000-fold lower inhibitory activity against TcG6PDH (IC_50_ > 50 µM). This clearly suggests that certain steroid analogues in vivo inhibit other essential molecular targets beyond G6PDH with a higher affinity.

With the exception of the un-substituted analogues (**EA**, **DHEA** and **7** = EC_50_ > 24–44 µM), all derivatives displayed a significantly higher cytotoxic effect against bloodstream *T. brucei* (EC_50_ 2–18 µM, 24 h incubation) than towards *T. cruzi* epimastigotes (estimated EC_50_ ≥ 50 µM, 72 h incubation). For *T. brucei*, compared to **EA** (EC_50_ = 36.5 µM) and **DHEA** (EC_50_ = 43.8 µM), the EC_50_ for compounds **1–3** and **4–6** was from 7- to 19-fold and from 2- to 8-fold higher, respectively. Several of the new analogues displayed a similar or higher potency than the control drug Nifurtimox (EC_50_ = 6.0 µM), although none of them reached the submicromolar potency of Conessine (EC_50_ = 0.42 µM) [44].

With respect to mammalian cells, except for **5** (CC_50_ = 39.0 µM) and **7** (CC_50_ > 24 µM), the remaining analogues also proved to be more cytotoxic than **EA** and **DHEA**. Notably, the most cytotoxic compounds from each class were the 3-acetoxy, 16β-brominated analogues 3 (CC_50_ = 0.96 µM) and 6 (CC_50_ = 4.5 µM) with CC_50_ values near 100- and 6-fold higher than those of EA and DHEA, respectively. In consequence, these compounds, as well as DHEA, proved the less selective against the pathogen. In contrast, compounds **1** (SI = 4.6) and **2** (SI = 8), and **4** (SI = 2.5) and **5** (SI = 2.2) showed similar or better selectivity than **EA** (SI > 2.7) and **DHEA** (SI = 0.6), respectively.

Besides the differences pointed above for the biological activity of the compounds, there was no correlation between inhibition of trypanosomal G6PDH and anti-trypanosomal or mammalian cytotoxicity for all **EA** and **DHEA** derivatives. Such behavior is particularly intriguing for compounds **2** and **5**, which displayed the highest in vitro selectivity towards the TcG6PDH enzyme. A possible explanation for this is that enzymatic hydrolysis of the acetoxy group (e.g., by esterases) affords the alcohol derivative at position 3, which may present a lower affinity for TcG6PDH. In contrast, similar products of the β-brominated derivatives **3** and **6** may render these compounds more active against human G6PDH or, eventually, may have other molecular targets.

### 2.6. Steroid Derivatives Affect the Intracellular Redox State of Bloodstream T. brucei

Several factors may account for the aforementioned lack of correlation between trypanosomal G6PDH inhibition in vitro and biological potency. Examples of these factors include compound uptake and metabolization/conversion, all of which may reduce the intracellular content of the active form of the original compounds. In some cases, the metabolized compounds may gain affinity for new molecular targets. In the absence of efficient NADPH^+^-synthesizing backup systems, inhibition of G6PDH should lead to a decrease in the NADPH^+^/NADP^+^ ratio. Among other functions, NADPH^+^ is required to maintain the intracellular pool of low molecular weight and protein thiols in reduced form [45]. On the other hand, inhibition of G6PDH has consequences on the downstream output of the PPP pathway, i.e., it causes a shortage of ribose 5-phosphate that impairs cell proliferation (the cytostatic effect). Thus, in order to verify the on-target effect of the steroid derivatives and their metabolic effect in the pathogen, we used a redox-reporter cell line of bloodstream *T. brucei* [46]. Based on the higher potency of the derivatives against bloodstream *T. brucei* and the fact that this parasite stage represents a clinically more relevant biological model to investigate compounds mode of action, the studies were performed on this parasite and not on *T. cruzi* epimastigotes.

The redox biosensor (hGrx1-roGFP2) [47] consists of a redox sensitive green fluorescent protein (roGFP2) fused to human glutaredoxin 1 (hGrx1) that enables a rapid redox equilibration between roGFP2 and the pool of reduced and oxidized glutathione and trypanothione [46,48,49].

Exponential phase redox-reporter parasites were treated with the steroid analogues added at 2× their EC_50_ (EC50), with Conessine at 10X its EC_50_ or with vehicle (1% *v*/*v* DMSO). From this study, we excluded all compounds showing poor anti-parasite activity (**EA**, **DHEA** and 7). The cells were incubated with compounds only 4 h prior to analysis, which is less than the doubling time for bloodstream *T. brucei* (6–7 h), to obtain a clearer picture of the cause-effect correlation at the onset of the treatment. As control treatments, parasites were shortly exposed to menadione or diamide (250 µM for 20 min), which consumes NADPH^+^ during redox cycling or is a potent thiol oxidizing agent [50], respectively.

**Figure 8:**
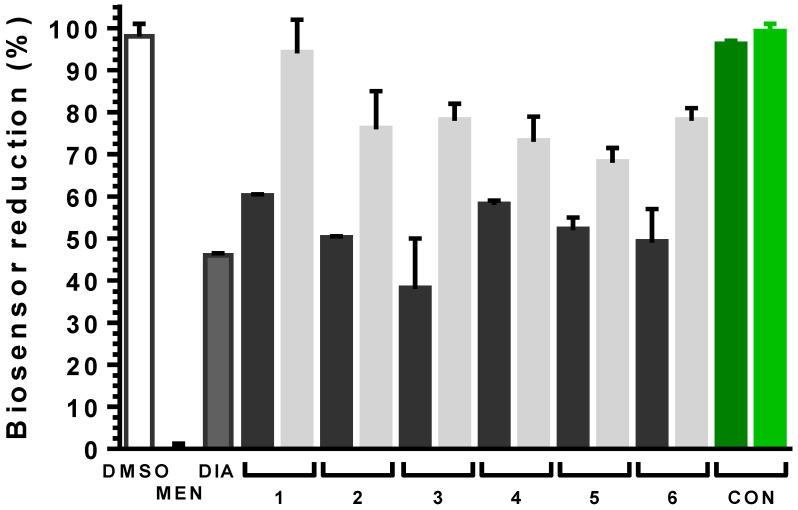
Intracellular redox changes induced by steroid derivatives in bloodstream *T. b. brucei*. Black bars, parasites treated with **EA** (**1**, **2** and **3**) or **DHEA** (**4**, **5** and **6**) derivatives at 2× their corresponding EC_50_ values (Table 2) for 4 h; Light gray bars, samples from the corresponding steroid-treated parasites exposed for 20 min to 1 mM DTT. The white and dark gray bars correspond to parasites treated with vehicle (1% *v*/*v* **DMSO**) for 4 h and with 250 μM menadiona (**MEN**) or diamide (**DIA**) for 20 min. The dark and light green bar correspond to parasites treated with 2 μM Conessine (**CON**, 5× EC_50_) for 4 h and, thereafter, with 1 mM DTT for 20 min, respectively. The % biosensor reduction was calculated normalizing the values to the conditions giving full biosensor reduction (parasites treated with 1% DMSO and DTT 1 mM) and oxidation (parasites treated with 250 μM menadione for 20 min) and is expressed as mean ±SD. Each experiment was carried out at least three times. Statistical analysis was performed applying a one-way ANOVA test with 95% confidence intervals with the Dunnet’s multiple comparisons test.

In infective trypanosomes, all steroid derivatives induced an oxidizing intracellular milieu as revealed by the significant 40 to 60% decrease in the relative amount of reduced biosensor compared to the vehicle control (*p* < 0.0001; Figure 8). Notably, the level of oxidation induced by steroids in a 4 h time-window was similar to that caused in 20 min by the strong thiol oxidizing agent diamide (54% biosensor oxidation) tested at a 6-60-fold higher concentration than the compounds. Importantly, to confirm the redox-basis of biosensor oxidation, steroid-treated parasites were exposed to the membrane permeant reducing agent dithiothreitol (DTT 1 mM) for 20 min. Although to different extent, but in all cases significantly (*p* < 0.0008), a short exposure to DTT was able to restore a more reducing environment in the compound-treated parasites (Figure 8, gray bars).

Although tested at concentrations that should exert a similar cytotoxic effect, the **EA** derivatives showed the following order of oxidizing potential: **1** < **2** < **3** (*p* < 0.02). A similar trend was observed for the **DHEA** analogues but the differences were not statistically significant.

Conessine has been previously reported as an inhibitor of trypanothione reductase and trypanothione syntethase [51]. Inhibition of these indispensable molecular targets should entail a significant intracellular redox unbalance [52,53,54]. However, in line with the observed lack of activity towards the trypanosomal G6PDH (Table 1), Conesine, at 5× its EC_50_ (or even 24× its EC_50_, not shown) did not induce oxidative stress in bloodstream trypanosomes (Figure 8). Taken together, this strongly suggests that this steroid alkaloid, at least in African trypanosomes, have a different mode of action and/or molecular target(s).

In summary, the results demonstrate that all **EA** and **DHEA** derivatives targeting in vitro TcG6PDH exert their toxic effects on trypanosomes, at least in part, through alteration of the thiol-redox homeostasis as consequence of a shortage on NADPH^+^ supply that compromises the maintenance of a physiologically high GSH/GSSG and trypanothione/trypanothione disulfide ratio.

## 3. Conclusions

In agreement with digitonin fractionation studies [24], our study confirmed by immuno-and cytochemical-based techniques that G6PDH is mostly a cytosolic and low abundant protein in the non-infective form of *T. cruzi*. The last finding is in agreement with the fact that amino acid catabolism is the major energy source for this parasite stage and that epimastigotes proved to be less susceptible to steroids than bloodstream *T. brucei*. In the extracellular stage of the parasites, which dwell in the host bloodstream, lymphatic or interstitial fluids (trypomastigote stage), the supply of reducing power is likely shifted towards the PPP. In these compartments, glucose content is higher than in the insect gut or salivary glands. This metabolic adaptation keeps pace with the fact that extracellular parasites should be prepared to face host oxidative challenge, while intracellular parasites are hidden in a reducing environment and protected from host-cell defenses. In line with this statement, the thiol-redox metabolism of bloodstream *T. brucei* appeared to be highly sensitive to G6PDH inhibition (see comments below).

The orientation of the Br atom occupying position 16 of **EA** and **DHEA** proved important in determining the potency of the steroids. The α-substituted derivatives presented higher activity than those presenting a β-orientation, which, based on structural analysis, could be ascribed to a more stable binding and steric effect on the catalytic site of the former. The inclusion of an acetoxy group at position 3 of 16α-**EA** and -**DHEA**, led to the identification of the most selective TcG6PDH inhibitors ever reported (almost three orders of magnitude). The increase in selectivity was mostly due to loss of affinity of the analogues for the human enzyme rather than to a gaining of affinity for the parasitic G6PDH.

The potential binding mode of steroids to host G6PDH has been disclosed and, despite being compatible with the kinetic mechanism of inhibition, proved to differ significantly from that of TcG6PDH. The structure adopted by a short lid-like element near the catalytic site and located in the dimerization domain of G6PDH seems key in determining the species-specific accessibility of steroids to the enzyme active site. Despite contributing to understanding the selectivity observed for steroids, the binding models might be valuable for the rational design of improved inhibitors against both enzymes.

In vitro inhibition of G6PDH by different **EA** and **DHEA** derivatives correlated with in-cell enzyme inhibition (in situ G6PDH activity) for *T. cruzi* epimastigotes and redox unbalance for bloodstream *T. brucei*. The remarkable intracellular oxidizing milieu induced by a short exposure to steroids highlights the key role of G6PDH in NADPH homeostasis and the lack of complementary and efficient backup systems for the synthesis of this metabolite in bloodstream parasites. Our results also indicate that steroids may have other important molecular targets in trypanosomes and mammalian cells. This is supported by the fact that some steroids lacking inhibitory activity against the *T. cruzi* (IC_50_ > 50 µM) or human G6PDH (IC_50_ > 100 µM) displayed a moderate to significant cytotoxicity towards epimastigotes or murine macrophages (EC_50_ 1–42 µM). Although G6PDH appears to be dispensable for *T. cruzi* epimastigotes, the killing potential of the steroids and the essentiality of this enzyme should be investigated in the clinically relevant forms of this parasite. Regarding African trypanosomes, G6PDH has been chemically validated here as a drug target.

## 4. Materials and Methods

### 4.1. Protein and Ligand Preparation

To model the binding pose of **EA** and its derivatives in the *T. cruzi* G6PDH, we used the enzyme in complex with glucose-6-phosphate and NADPH (PDB ID: 5AQ1) as a starting point and then we modeled the oxidized form of the cofactor by: (i) oxidizing the NADPH to NADP+ (NAP) in place followed by energy minimization (model 1); (ii) extracting the oxidized form of the cofactor from the human coordinates 2BH9 (model 2) and aligning them onto the 5AQ1 coordinates.

Both models were prepared with the Protein Preparation Wizard [55] in order to add missing H atoms, to assign the correct ionization state of both substrate and cofactor at physiological pH using Epik [56,57] and to assign the proper ionization to the amino acids using PROPKA.

For the human G6PDH, we used the PDB coordinates 6E08, representing the enzyme in complex with structural NADP+. The coordinates of the substrate glucose 6-phosphate and of the catalytic NADP+ from the human PDB coordinates 2BHL and 2BH9, respectively, were copied. The full model was then prepared with the Protein Preparation Wizard as well.

**EA** and its derivatives were prepared using LigPrep [58].

### 4.2. Binding Site Detection and Docking

We performed a binding site analysis of T. cruzi G6PDH model 1 and model 2, and of the human G6PDH model using SiteMap [59,60,61] at default settings. The model 1 of TcG6PDH consisted of the coordinates for the enzyme co-crystallized with NADPH [37] computationally oxidized to NADP^+^ (NAP) and minimized; for model 2, the atomic coordinates of NAP^+^ were extrapolated from the structure of the co-crystallized human enzyme (PDB 2BH9) and aligned onto the TcG6PDH structure (PDB 5AQ1), since the G6P and NADP sites are highly conserved in both species. A docking study was performed on all the 3 models using Glide [62,63,64,65]. For each model, the Glide grid was centered on the highest scored binding site according to the SiteMap Dscore. The Dscore (druggability score) is used to distinguish a “druggable” biding site from an “un-druggable” or “difficult” binding site using the weighted sum of several properties as follows [60,65]:

Dscore = 0.094 sqrt(n) + 0.60 e − 0.324 p.

where n is the number of site points, e is the enclosure score and p is the hydrophilic score. Usually, Dscores with values of 1 and above are considered to be “druggable”.

The Glide docking was performed using the Standard Precision scoring function (Glide SP), at default settings saving 3 poses per compound.

For the analysis of the binding mode of steroids to TcG6PDH, we chose model 2 because: (i) the binding mode of the cofactor in human G6PDH (PDB 2BH9) was already co-crystallized in the oxidized form that was of interest to us; (ii) as mentioned above, the catalytic site in *T. cruzi* and human G6PDH is highly conserved; (iii) the predicted binding pose of **EA** and its derivatives in model 2 is in better agreement with the experimental evidence.

### 4.3. Reagents

Chemical reagents were of analytical or higher grade and obtained from Sigma-Aldrich or AppliChem. Antibiotics were purchased from Invitrogen and Sigma-Aldrich. All protein purification resins and columns were from General Electric Healthcare Life-Sciences (GE). The media and the consumables for cell cultures were purchased from Invitrogen and Greiner, respectively.

The chemical syntheses of the compounds were published in [27] and in [44].

### 4.4. Expression and Purification of Recombinant Proteins

*E. coli* Tuner (DE3) cells were transformed with the pET28a (+) and pHis constructs encoding for N-terminally hexa histidine-tagged TcG6PDH_L_ and HsG6PDH, respectively, and grown overnight in LB medium with 50 µg/L kanamycin and 100 µg/L ampicillin, respectively. ZYM-5052 auto-induction medium with 100 µg/L kanamycin or ampicillin was inoculated at a ratio of 1:100 with overnight cultures and incubated for 4 h at 37° and 220 rpm. Thereafter, the cultures were incubated for 20 h at 25 °C and 170 rpm. Cells were harvested by centrifugation (5000× *g*, 10 min, 4 °C), resuspended in buffer A (50 mM Tris, pH of 8.0, 500 mM NaCl and 5 mM MgCl_2_) containing a complete™ Protease Inhibitor Cocktail prepared as indicated by the provider (Sigma-Aldrich-Merck, Germany); afterwards, we added 1 mg/mL of lysozyme and incubated the cells for 1 h at 4 °C. Cells were further lysed by three cycles of sonication (45% amplitude, 2 s pulse on/off, for 1 min) using a macrotip in a Branson Digital Sonifier 450 (Marshall Scientific, Hampton, VA, USA) and debris was removed by centrifugation at 27,000× *g* for 45 min at 4 °C and filtrated through a 0.45 µm filter (Millipore-Merck, Darmstadt, Germany). The cleared lysate was loaded onto a HisTrap column pre-equilibrated with buffer A. The column was washed with 10 and 5 column volume of buffer A and 5 mM imidazol in buffer A, respectively, and finally the His-tagged proteins eluted with 500 mM imidazol in buffer A. The fractions containing the recombinant protein were collected, concentrated via ultrafiltration at 4 °C, 7000× *g* (10-kDa filter cutoff) and gel filtrated on a Superdex 200 10/300 GL column pre-equilibrated with buffer A. Fractions containing the protein of interest, as assessed by Coomasie blue stained SDS 10% PAGE gels, were collected and tested for enzyme specific activity. Protein concentration was determined from the absorbance values at 280 nm and the corresponding theoretical absorbance coefficients.

### 4.5. G6PDH Enzymatic Assay

G6PDH activity was determined at room temperature (RT) by monitoring NADPH (ε_340_ = 6220 M^−1^ cm^−1^) formation at 340 nm. All reactions were performed in 50 mM Tris (pH 7.5) with 5 mM MgCl_2_ and 250 mM NaCl in a final volume of 500 µL. The reactions were started by the addition of 700 µM NADP^+^ and 6 mM G6P for TcG6PDH or by the addition of 250 µM NADP^+^ and 1 mM G6P for the human enzyme. For each enzyme, a control reaction in the presence of 1% (*v*/*v*) DMSO was run. Stock and working solutions of compounds were prepared in 100% (*v*/*v*) DMSO. For IC_50_ determinations, at least seven different concentrations of compounds were tested (range: 0.002–50 µM). The initial velocities were calculated from the first 45 s of the reaction and normalized to the corresponding enzyme control. All measurements were performed at least in triplicate using a Cary 50 Bio spectrophotometer (Agilent Technologies, Santa Clara, CA, USA) at RT. The initial rates were determined with the Origin Pro 8.0 software (OriginLab Corporation, Northampton, MA, USA), and the IC_50_ values were calculated by nonlinear regression using the GraphPad Prism 5.0 software (GraphPad Inc., San Diego, CA, USA).

### 4.6. Generation of T. cruzi Cell Line with Inducible Expression of G6PDH

Epimastigotes from *T. cruzi* strains Adriana [66] were grown axenically at 28 °C in brain-heart tryptose medium (BHT; [67] supplemented with 2 g/L glucose, streptomycin (100 μg/mL), penicillin (100 IU/mL), hemin (20 μg/mL), and 10% (*v*/*v*) heat-inactivated tetracycline-free fetal bovine serum (FBS; PAA, Houston, TX, USA). For inducible expression of *Tc*G6PDHL in *T. cruzi* strain Adriana, we first generated a cell line expressing the T7 RNA polymerase and tetracycline repressor genes by transforming the parasites with the plasmid pLew13 [68]. Epimastigotes from *T. cruzi* in the mid-log phase were washed with and resuspended in BHT medium at a final concentration of 5 × 10^8^ parasites/mL. Aliquots of 0.35 mL were dispensed into disposable 0.4-mm cuvettes (Bio-Rad Laboratories, Hercules, CA, USA) containing 10 μg of plasmid DNA and electroporated using a Bio-Rad gene pulser at 335 *V* and 1400 mF, with two consecutive pulses. After 5 min on ice, the parasites were diluted 10-fold with BHT containing 10% FBS and allowed to recover for 24 h at 28 °C before adding 200 μg/mL of Geneticin (G418; Life Technologies, Carlsbad, CA, USA). Cells stably transfected with pLew13 were then electroporated with the p*Tc*INDEX-*Tc*G6PDH_L_ construct and transgenic parasites were obtained after 3 weeks of selection with 200 μg/mL G418 and 200 μg/mL hygromycin B (Calbiochem-Merck, Darmstadt, Germany) [69]. The expression of the ectopic copy of *Tc*G6PDHL was induced by growing the parasites in culture medium containing 5 or 10 μg/mL oxytetracycline (Laboratorios Microsules, Montevideo, Uruguay).

### 4.7. Western Blot

Anti-*Tc*G6PDHL serum was raised in mice (strain Balbc/J) against the purified recombinant *Tc*G6PDHL using a standard immunization protocol approved by the Animal Use and Ethic Committee (CEUA) of the Institut Pasteur Montevideo (Protocol n° 005-14) and animals from the in-house specific pathogen free animal facility (Transgenic and Experimental Animal Unit, Institut Pasteur de Montevideo). A total of 10,000,000 to 200,000,000 epimastigotes in the late exponential phase were harvested by centrifugation at 2000× *g* for 10 min at RT and washed twice with phosphate-buffered saline (PBS, pH 7.4), incubated for 15 min in 10 mM Tris-HCl, 1 mM EDTA, 0.5% (*v*/*v*) Triton X-100 at 4 °C and then centrifuged at 13,000× *g* for 30 min at 4 °C; afterwards, a SDS-PAGE loading buffer (30 mM Tris HCl pH 6.6, 1% (*w*/*v*) SDS and 5% (*v*/*v*) glycerol) was added to the supernatant followed by two freezing-thawing steps [70]. Proteins from total cell extracts of tet-induced and non-induced parasites and different amounts of recombinant *Tc*G6PDHL were separated by SDS-10% PAGE under reducing conditions and transferred to a polyvinylidene fluoride (PVDF) membrane (GE-Healthcare, Chicago, IL, USA). The membrane was blocked with PBS-0.2% (*v*/*v*) and Tween-20 (PBS-T). We then added 5% (*w*/*v*) non-fat dry milk overnight at 4 °C, washed the membrane with PBS-T and incubated with polyclonal mouse serum anti-*Tc*G6PDHL diluted 1:1000 in PBS-T at RT for 2 h. After three washing steps with PBS-T for 5 min each, the membrane was incubated for 1 h at RT with the corresponding horseradish peroxidase-conjugated goat anti-mouse IgG (Amersham Biosciences, Buckinghamshire, England) as the secondary antibody at a 1:10,000 ratio in PBS-T. The membrane was extensively washed in PBS-T prior to chemoluminiscent detection of reactive bands with the Amersham ECL™ Western Blotting Detection Reagents (GE Healthcare) and according to the manufacturer’s instructions. Finally, films (Amersham hyperfilm ECL, GE Healthcare) were exposed to the membranes during different times (1, 5 and 10 min) and were revealed manually for 1 min in the developing solution (Sigma-Aldrich-Merck, Darmstadt, Germany).

### 4.8. Indirect Immunofluorescence

Five million *T. cruzi* epimastigotes (strain Adriana) induced or not to express an ectopic copy of G6PDH_L_ and grew to the late exponential phase in the absence or presence of 10 μg/mL oxytetracycline were pelleted at 2000× *g* for 10 min at RT and washed twice with PBS. For fixation, the cells were resuspended in PBS with 4% (*w*/*v*) paraformaldehyde, incubated for 18 min at RT, washed twice with PBS and resuspended in PBS at ~1 × 10^5^ parasites/μL. Drops containing about 50 μL of this cell suspension were added to a polilysine treated glass slide (StarFrost, Knittel Glass, Braunschweig, Germany) and incubated overnight at 4 °C. The cells were then permeabilized with 0.2% (*v*/*v*) Triton X-100 in PBS for 20 min and washed twice with PBS. The slides were incubated at 4 °C overnight and 1 h with purified mouse polyclonal anti-*Tc*G6PDH (1:1000), anti-LipDH (1/200) and rabbit anti- *Tc*TR (1:500), respectively, prepared in PBS added of 0.5% (*w*/*v*) gelatin. Then, the slides were washed with PBS and incubated for 1 h with the secondary antibody Alexa Fluor^®^ 488-labeled goat anti-mouse IgG and Alexa Fluor^®^ 594-labeled goat anti-rabbit IgG, respectively, both of which were added at a final dilution of 1:1000. Nucleic acids were stained with Hoechst 3342 (Invitrogen, Carlsbad, CA, USA) and cell membranes with CellMask Red (Thermofisher). The parasites were visualized with a Leica TCS SP5 spectral confocal microscope and the merge images were generated with the program Adobe Photoshop.

### 4.9. Cytochemical Assay for G6PDH Enzymatic Activity

The inhibition of G6PDH by epiandrosterone (**EA**) was addressed using non-infective epimastigotes from *T. cruzi* strain CL-Brener and DM-28c, and an optimized protocol previously described for cytochemical detection of G6PDH activity in mammalian cells [43]. About 5 × 10^6^ cells from an exponential phase culture were treated with 0, 25 and 100 µM EA for 24 h, then were pelleted at 1800× *g* for 10 min at RT and washed three times with PBS. The cell suspension was then incubated with 20 mM NADP^+^ in buffer B (Tris 50 mM pH 7.5, 5 mM MgCl_2_) for 20 min at 4 °C. The cells were fixed in 4% (*w*/*v*) paraformaldehyde for 30 min at RT, washed twice with 0.2% (*v*/*v*) Triton X-100 in PBS and twice with PBS. After each washing step the cells were pelleted by centrifugation at 1800× *g* during 5 min at RT. The permeabilized cells were resuspended in 100 μL of buffer B, added to a 600 μL reaction buffer (10 mM G6P, 800 μM NADP^+^, 0.3 mM phenylmethanesulfonyl fluoride, 5 mM sodium azide, 5 mM cyano-ditolyl tetrazolium chloride and 6% (*w*/*v*) of polyvinyl alcohol, all prepared in buffer B) and incubated for 30 min at RT. The reaction was stopped with 1 mL PBS, followed by three washes in PBS and centrifugation at 1000× *g* for 3 min at RT. Finally, cells were resuspended in PBS at a density of 1 × 10^5^ cells/mL and 50 μL of this suspension was added to a polylysine coated microscope slide (StarFrost, Knittel Glass, Germany), followed by incubation overnight at 4 °C. Nucleic acids were stained with DAPI (Fluoroshield Histology Mounting Media, Sigma-Aldrich-Merck, Germany). Cell images (bright field and fluorescence) were obtained with a confocal microscope Leica TCS SP5 and further analyzed and prepared with the program ICy [71] and Adobe Photoshop CS5.

## 5. Cell Viability Assays

*T. cruzi* epimastigotes (strain DM28) were grown at 28 °C in LIT medium supplemented with 10% (*v*/*v*) Fetal Bovine Serum (FBS; GIBCO^®^), 7.5 mM hemin and antibiotics (penicillin 10 U/mL– streptomycin 10 µg/mL). To determine parasite viability, 5 × 10^5^ parasites/mL in mid- exponential grow phase were seeded in a 96 well-plate (200 µL of the cell suspension/well) containing 2 µL of steroid derivatives at different concentrations (1, 10 and 50 µM), or 2 uL of vehicle DMSO or Nifurtimox (24 uM) and incubated during 72 h at 28 °C. Thereafter, 200 µL from each well were transferred to individual cytometer tubes. Propidium iodide (exclusion dye; PI) was added at a final concentration of 2 µg/mL and all samples were analyzed with a BD FACSAria™ Fusion Cell Sorter (laser/filter pair: λex/em = 561 nm 695 ± 20 nm for PI). The data were processed and analyzed with the FlowJo-V 10.7.1 software.

Bloodstream *T. b. brucei* (strain 427- cell line 514-1313) constitutively expressing an ectopic copy of the luminescent red-shifted biosensor LUC [72] was grown in HMI-9 medium complemented with 10% (*v*/*v*) Fetal Bovine Serum Tetracycline-free (FBS; GIBCO^®^, Thermo-Fisher Scientific, Waltham, MA, USA) in a humidified incubator with 5% CO_2_ and at 37 °C. Parasite viability was performed as described in [72]. Briefly, 1 × 10^5^ parasites/mL in a mid-exponential growth phase were seeded in a 96 well-plate (200 µL of the cell suspension/well) containing 2 µL of steroid derivatives at different concentrations (range: 0.035–50 µM), and incubated over 24 h. Thereafter, 200 µL from each well were transferred to a 96-well black plate and incubated with 20 µL of a solution containing: 1.5 mg/mL luciferin in PBS glucose 1% (*w*/*v*) and Triton X-100. The bioluminiscent signal was measured for 5 s at 37 °C in a luminometer (LUMI star OPTIMA microplate) over every 8 min period for a total of 32 min.

Murine macrophages (cell line J774) were cultivated in DMEM medium supplemented with 10% (*v*/*v*) FBS, 10 U/mL penicillin and 10 µg/mL streptomycin, under a humidified 5% CO_2_/95% air atmosphere at 37 °C. Cell viability was assessed using the WST-1 reagent. For CC_50_ determination (24-well culture plate), macrophages (6.25 × 10^4^ cells/mL × well) were incubated with different steroid concentrations (range: 0.6–75 µM) during 24 h at 37 °C.

For all assays, each condition (controls and compounds) was tested in triplicates and cell viability was calculated as follows: viability (%) = 100% × (number of cells for compound Y at concentration X/number of cells in the DMSO-treated control). EC_50_ values were obtained from the corresponding concentration/response plots fitted to a sigmoidal Hill equation or extrapolated from nonlinear fitting equations. The errors were calculated using error propagations and are expressed as S.D. estimated as σ^(n−1)^.

### Redox Reporter Assay

Bloodstream *T. b. brucei* expressing the redox biosensor hGrxroGFP2 was grown as described elsewhere [46]. Briefly, 2 × 10^6^ cells/ mL were seeded per well in a 96-well plate and incubated with different steroids derivatives each at 2× their EC_50_, 250 µM menadione, 250 µM diamide or 1% *v*/*v* DMSO for 4 h (5% CO_2_ and 37 °C). Next, 50 µL from each well were transferred to a 96-well plate, containing 100 µL of sterile PBS with glucose 1% (*w*/*v*). For each condition tested, a second sample was incubated with 1 mM DTT 20 min prior to analysis by flow cytometry, while propidium iodide (exclusion dye; PI) was added at a final concentration of 2 µg/mL All samples were analyzed with a C6Accuri flow cytometer (BD) as described in [46]. GFP fluorescence (filter λem = 530/40 nm) was only analyzed for viable cells (PI negative). The data were processed and analyzed with the C6Accuri software. Samples were analyzed in triplicate and the error is expressed as S.D.

## Figures and Tables

**Table 1:** Inhibition of G6PDH by (dehydro)epiandrosterone derivatives.

	IC_50_ (µM)		
Compound	*T. cruzi*	*H. sapiens*	Selectivity Index ^b^
**EA**	3.0 ± 0.4 ^c^	9.4 ± 1.0 ^a^	3
**1**	0.053 ± 0.002	20.8 ± 4.4 ^a^	392
**2**	0.11 ± 0.02	>100	>909
**3**	1.5 ± 0.2	>100	>67
**DHEA**	21.5 ± 0.5 ^d^	11.3 ± 2.2 ^a^	0.5
**4**	3.3 ± 0.7	48.0 ± 2.6	14
**5**	0.079 ± 0.01	>100	>1266
**6**	>50	>100	>2
**7**	>50	15.8 ± 3.3 ^a^	0.32
Conessine	>100	>100	ND

^a^ IC_50_ values reported in [27]; ^b^ selectivity index calculated as IC_50_ for human G6PDH vs. IC_50_ for *T. cruzi* G6PDH; Data reported in ^c^ [33] or ^d^ in [14].

**Table 2:** Biological activity of (dehydro)epiandrosterone derivatives.

Compound	% Cytotoxicity (50 µM) ^a^	EC_50_ (µM) ^a^	CC_50_ (µM) ^a^	Selectivity Index ^b^
	*T. Cruzi* Epimastigote Form	*T. brucei* Bloodstream Form	Murine Macrophage (Cell Line J774)	
**EA**	3.7 ± 0.7	36.5 ± 3.6 ^c^	>100	>2.7
**1**	14.5 ± 5.3	1.9 ± 0.2	8.8 ± 1.0	4.6
**2**	64.0 ± 4.1	5.3 ± 0.4	42.0 ± 1.0	7.9
**3**	73.9 ± 3.4	4.8 ± 0.5	1.0 ± 0.1	0.2
**DHEA**	n.d.	43.8 ± 2.0 ^c^	25.0 ± 13.0 ^d^	0.6
**4**	6.4 ± 1.6	7.5 ± 0.3	19.0 ± 3.0	2.5
**5**	30.9 ± 2.5	18.0 ± 3.0	39.0 ± 3.0	2.2
**6**	76.2 ± 0.8	5.3 ± 0.5	4.5 ± 0.4	0.9
**7**	3.9 ± 0.7	>24	>24	n.d
Conessine	n.d.	0.42 ± 0.01 ^e^	61.2 ^e^	145.7
Nifurtimox	46.0 ± 1.9 (at 24 µM)	6.0 ± 0.4	140.0 ± 2.0	23.3

^a^ Data presented as mean value ± standard deviation of at least three independent determinations. ^b^ The selectivity index (SI) was calculated as the ratio: CC_50_ for murine macrophages/EC_50_ for *T. brucei*. ^c^ value reported by [44]. ^d^ value reported against Hek293T by [27]. ^e^ Values against mammalian L6 cell line from rat skeletal myoblasts reported by [44]. n.d. not determined.

## Data Availability

not applicable.

## Supplementary Materials

The following are available online, Figure S1: In situ detection of G6PDH activity in *T. cruzi* strain Adriana overexpressing G6PDH, Figure S2: In situ detection of G6PDH activity in *T. cruzi* strain Dm28c.

## Sample Availability

Samples of the compounds are not available from the authors.
