# Supplementary material for: Glucose 6-Phosphate Dehydrogenase from Trypanosomes: Selectivity for Steroids and Chemical Validation in Bloodstream Trypanosoma brucei
Source: Molecules. 2021 Jan 12;26(2):358. doi: 10.3390/molecules26020358 (PMC7826790; doi:10.3390/molecules26020358)
Supplement: Supplementary file 1 [file molecules-26-00358-s001.pdf]

molecules

# Glucose 6-phosphate dehydrogenase from trypanosomes: selectivity for steroids and chemical validation in bloodstream *Trypanosoma brucei*

**Cecilia Ortíz, Francesca Moraca, Marc Laverriere, Allan Jordan, Niall Hamilton and Marcelo A. Comini**

## Supplementary Information

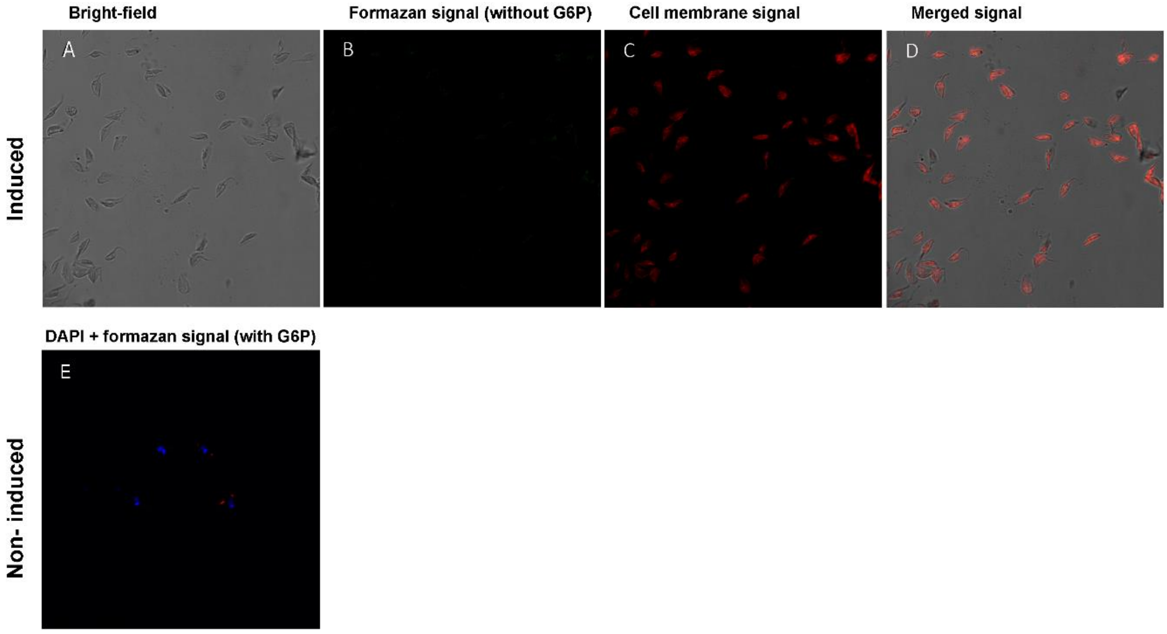


**Figure S1. *In situ* detection of G6PDH activity in *T. cruzi* strain Adriana overexpressing G6PDH.** Epimastigotes from *T. cruzi* strain Adriana grown for 24 h in the absence (Non-induced) or the presence of 10 µg/mL oxytetracycline (Induced) to induce overexpression of *Tc*G6PDHL. Negative control of the NADPH-dependent reduction of 5-cyano-2,3-ditolyl-tetrazolium chloride, the reaction was performed in absence of the substrate G6P. For oxytet-induced samples: **A**) Bright field image, **B**) cytochemical reaction performed in the absence of the enzyme substrate glucose 6-phosphate (G6P), **C**) cell membranes stained with CellMask and **D**) Merge images. **E**) For oxytet non-induced sample is shown the merge of the DAPI and formazan signal performed in the presence of the enzyme substrate G6P.

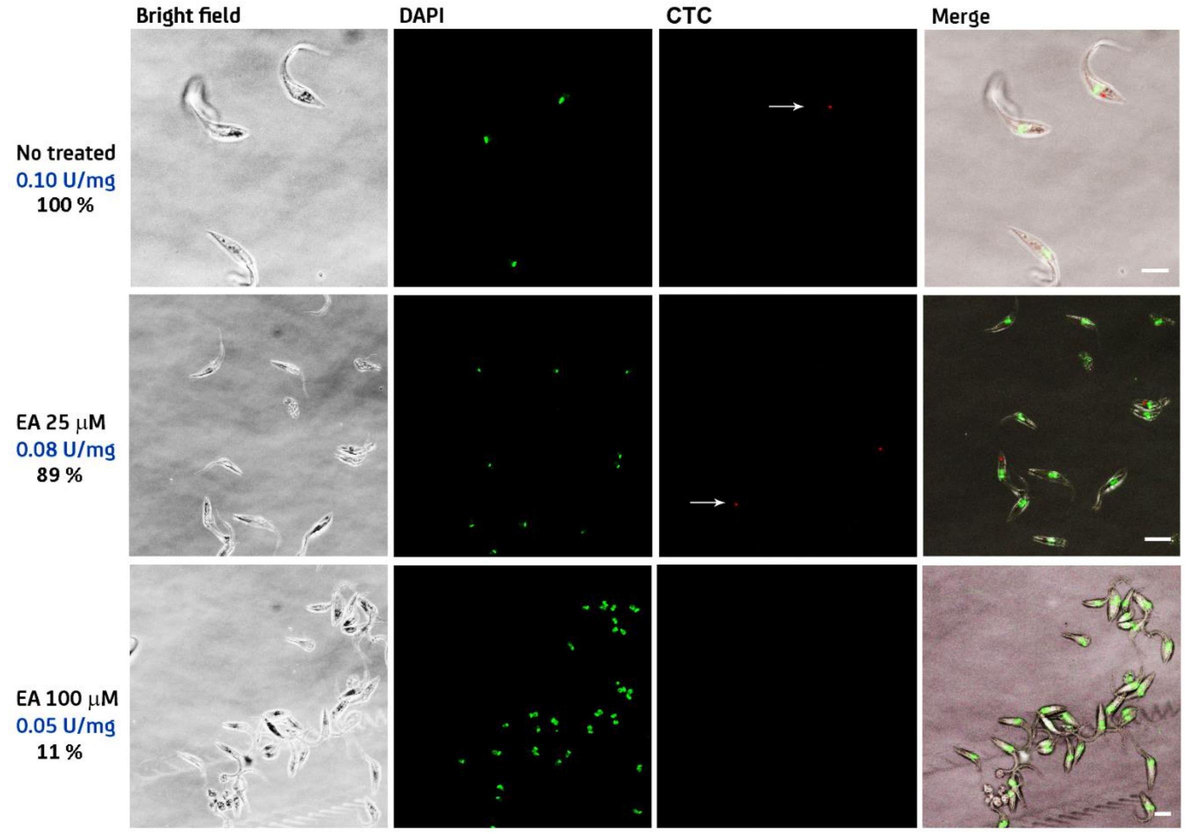


**Figure S2.** ***In situ*** **detection of G6PDH activity in** ***T. cruzi*** **strain Dm28c.** Epimastigotes were incubated for 24 h in the absence (no treated) or presence of 25 and 100 μM epiandrosterone (EA). G6PDH activity was measured in cell extracts using the standard enzyme assay (specific activity expressed as U/mg) and detected at intracellular level using a couple cytochemical assay based on the reduction of 5-cyano-2,3-ditolyl-tetrazolium chloride (CTC; red color). For each condition tested, CTC positive cells are shown as percentage relative to non-treated parasites. Hoechst 3342 stain (green color) was used to mark nuclear and mitochondrial DNA. Bright field and merged images are also shown with the white bar indicating a length of 10 μm.
